# Data Augmentation for Deep-Learning-Based Multiclass Structural Damage Detection Using Limited Information

**DOI:** 10.3390/s22166193

**Published:** 2022-08-18

**Authors:** Kyle Dunphy, Mohammad Navid Fekri, Katarina Grolinger, Ayan Sadhu

**Affiliations:** 1 Department of Civil and Environmental Engineering, Western University, London, ON N6A 3K7, Canada; 2 Department of Electrical and Computer Engineering, Western University, London, ON N6A 3K7, Canada

**Keywords:** Structural Health Monitoring, deep learning, Generative Adversarial Networks, data augmentation, damage detection

## Abstract

The deterioration of infrastructure’s health has become more predominant on a global scale during the 21st century. Aging infrastructure as well as those structures damaged by natural disasters have prompted the research community to improve state-of-the-art methodologies for conducting Structural Health Monitoring (SHM). The necessity for efficient SHM arises from the hazards damaged infrastructure imposes, often resulting in structural collapse, leading to economic loss and human fatalities. Furthermore, day-to-day operations in these affected areas are limited until an inspection is performed to assess the level of damage experienced by the structure and the required rehabilitation determined. However, human-based inspections are often labor-intensive, inefficient, subjective, and restricted to accessible site locations, which ultimately negatively impact our ability to collect large amounts of data from inspection sites. Though Deep-Learning (DL) methods have been heavily explored in the past decade to rectify the limitations of traditional methods and automate structural inspection, data scarcity continues to remain prevalent within the field of SHM. The absence of sufficiently large, balanced, and generalized databases to train DL-based models often results in inaccurate and biased damage predictions. Recently, Generative Adversarial Networks (GANs) have received attention from the SHM community as a data augmentation tool by which a training dataset can be expanded to improve the damage classification. However, there are no existing studies within the SHM field which investigate the performance of DL-based multiclass damage identification using synthetic data generated from GANs. Therefore, this paper investigates the performance of a convolutional neural network architecture using synthetic images generated from a GAN for multiclass damage detection of concrete surfaces. Through this study, it was determined the average classification performance of the proposed CNN on hybrid datasets decreased by 10.6% and 7.4% for validation and testing datasets when compared to the same model trained entirely on real samples. Moreover, each model’s performance decreased on average by 1.6% when comparing a singular model trained with real samples and the same model trained with both real and synthetic samples for a given training configuration. The correlation between classification accuracy and the amount and diversity of synthetic data used for data augmentation is quantified and the effect of using limited data to train existing GAN architectures is investigated. It was observed that the diversity of the samples decreases and correlation increases with the increase in the number of synthetic samples.

## 1. Introduction

In recent years, developments within the field of Structural Health Monitoring (SHM) has focused on the integration of Artificial Intelligence (AI) techniques with visual inspection for autonomous structural maintenance [1]. The necessity for this advancement in this field arises from the challenges of traditional human-based structural inspections. Noncontact sensing technology [2] has shown significant promises to make SHM real-time and robust. However, extracting vision-based data from structures using cameras and other optical devices is often time-consuming and has significant economic costs [3,4,5,6,7]. Logistical restraints of the structures themselves or health hazards posed by structures damaged by natural disaster events may restrict access, making it difficult to complete the inspection. Even once data collection is completed, there are still challenges (e.g., damage categorization and determining the level of severity) in processing a large amount of vision-based data for SHM. The data collected from the structure is not easily analyzed and is a time-consuming process for engineering firms. In addition, human-based assessments often are biased, as the analysis of the data is based on a subjective opinion of the inspector [8,9]. This paper explores a novel data augmentation technique to address the data scarcity issue of SHM.

The autonomous and flexible nature of AI techniques has allowed researchers to rectify the issues (e.g., prolonged inspection times and biases in damage categorization) prevalent within the SHM field. In particular, a subset of AI techniques, namely convolution-based deep-learning techniques such as Convolutional Neural Networks (CNNs) have been well researched for damage assessment of concrete structures [10,11,12,13]. Applications of these algorithms for classification of image-based [14], region-based [15,16], and pixel-level [17,18] vision data have resulted in highly accurate and robust assessment techniques. Additionally, studies have been conducted for detection of various categories of cracks within asphalt pavement [19,20,21]. Transverse pavement cracks were identified using a hybrid time-frequency enhanced CNN based on Short-Time Fourier Transform and Wavelet Transform [19]. The inclusion of spectrograms resulted in high classification accuracies of 97.2% and 91.4%. respectively. Contrasting traditional approaches, these techniques directly learn from vision-based data and classify objects from extracted features of input data. Model parameters are updated through an iterative backpropagation method, meaning that direct human intervention to define and optimize model parameters is removed. Furthermore, they can be directly integrated with robotic platforms such as unmanned aerial vehicles, allowing for the remote inspection of structures [19,20], providing an economic, safe, and time-efficient method for SHM.

AI models, such as CNNs, are “Big Data” models, implying that the performance of these models is directly correlated to the amount of data available, usually >10,000 images for each class [3,7,8,9,22,23,24]. However, it is often difficult to collect vision-based data from damaged structures due to logistic restraints, and subsequent damage-causing events compound this issue. Structural damages are often a result of long-term fatigue, vehicle-based impact events, or natural disasters which are rare and infrequent, resulting in a reduced amount of data available for certain damaged cases [5,8,25,26]. Therefore, the data collected from structures is often heavily imbalanced and limited, with considerably more data being available in the “undamaged” or normal class when compared to the damaged classes. Training AI models on imbalanced datasets has been demonstrated to create biases during the learning process, resulting in a model whose performance is class-dependent rather than consistent across all classes [3,24]. Limited datasets result in poor model robustness as the models tend to experience overfitting based on the “noisy” features from the data rather than learning the general features associated with the particular class. Moreover, the lack of publicly available datasets within the SHM domain makes it difficult to train new, and highly accurate AI models [3].

To address these data scarcity and class imbalance issues, data augmentation has been proposed as a technique to enhance and equalize datasets used for training AI models [27]. In SHM, simple data augmentation techniques such as rotation, shear, zoom, mirroring, flipping, and contrast changes have been predominately used to enhance image-based datasets of structural damage [28,29,30]. Vertical and horizontal flipping, contrast adjustment, and rotation were applied [28] to augment an existing dataset for the binary classification of concrete cracks using pre-trained deep learning models. Similar transformations were applied [29] to enhance the number of segmented images of cracks of welded joints located on the gusset plates of steel bridges. The use of synthetic images during training increased the accuracy of the investigated classifier by 2–5%. An interpolation-based data augmentation technique was employed [31] to enhance a thermal image dataset for adversarial-based classification. These synthetic samples are then used to augment the existing dataset by balancing the classes and providing increased samples for training resulting in an AI model that is more accurate and robust than would be obtained on the original data.

Recently, there has been a significant debate on the effectiveness of simple data augmentation for addressing the data scarcity and class imbalance issues in SHM [22,24]. Due to the limited number of samples that comprise these datasets, the diversity of the features contained within the images that relate to the given class is relatively low. Simple data augmentation techniques such as flipping, rotating, and shifting create synthetic images whose features are only slightly different than those of the original images; therefore, the overall diversity of the dataset experiences a minimal change. Training an AI algorithm with low diversity datasets, even with the inclusion of simple synthetic images, often results in poor generalization and overfitting during training, which can result in lower performance than when trained entirely on real data [9]. To address the quality and diversity of synthetic image samples, Generative Adversarial Models (GANs) [32] have been widely explored by researchers in various domains for data augmentation problems. The basis of these models involves competition in a “zero-sum” game between two models (1) the ‘Discriminator’ which distinguishes between real and synthetic samples and (2) the ‘generator’ which generates synthetic images from a random Gaussian vector. Since its inception, various improved GAN models have been proposed, including conditional GANs [33], Wasserstein GANs [34], Wasserstein GANs with Gradient Penalty [35], and StyleGAN [36].

In SHM, GANs have been implemented to address two types of data augmentation problems, (1) synthesizing missing data points due to damage or poor quality sensors, and (2) expanding limited or unbalanced datasets for AI algorithm training. A deep convolutional GAN was applied [6] to reconstruct lost data from faulty bridge sensors. The experiments demonstrated that the proposed method was capable of accurately capturing the details of low- and high-frequency components of the signals with 0.5–4% error between real and synthetic samples for the first five mode shapes. However, the magnitude of the reconstruction error is heavily influenced by the number of faulty sensors used during instrumentation. Similarly, it was observed that increasing the number of faulty sensors from 5 to 15 increased the error within the simulated data from 3.7% to 9.44% when applying a traditional GAN structure for incomplete data extracted from long-term bridge monitoring [37]. The reconstruction error was noticeably correlated with the length of the time period of the synthetic data, where longer time periods (>8 h) had higher errors. A transfer learning-based GAN-autoencoder ensemble was implemented [38] for the detection of anomalies arising from faulty signal data for SHM applications. The signals recorded during monitoring were converted to images using a Gramian Angular Field and Cumulative Sum thresholding was applied to define the limits of the anomalies. Though this method demonstrated significant accuracy (>94%) for most signals, it was demonstrated that single-point signal anomalies may not be captured.

The more common application of GANs for data augmentation within SHM involves synthesizing images to expand existing datasets to rectify issues pertaining to limited data or imbalanced classes. Typically, the majority of SHM datasets consist of only a few hundred images with a heavy imbalance toward the normal (undamaged) state rather than the damaged cases which are more important to investigate. For instance, the original dataset implemented [25] contained 5900 disjoint, 3000 obstacles, 2600 walls, and 6390 tree root images before data augmentation occurred. Moreover, class imbalance ratios in the original datasets ranging from 32 to 2 have been observed for binary and multiclass problems [5,22,25]. Training AI algorithms using limited or unbalanced datasets results in low generalizing models that have poor accuracy and are biased to the class that has more data [26]. The hypothesis is that by creating synthetic samples using GANs to augment these datasets, the performance of the AI-based classification should improve. For instance, as a result of an expanded dataset produced by a GAN-based implanting technique, an increase of 18% in F1 score was observed when implementing a Faster R-CNN for construction resource classification [3]. Similar improvements were experienced [8] when applying progressively growing GANs to improve the pixel-level segmentation and classification of cracked images. Overall, the mean intersection over union score was increased by 50% through the inclusion of synthetic images and demonstrated improved performance over traditional data augmentation techniques such as scaling, translation, and rotation. Increased network performance was also observed [7] when applying a super-resolution GAN for road damage detection. Generally, the application of the proposed data augmentation regime improved the average classification by 1–3%. Moreover, it has been demonstrated that the addition of synthetic data has improved generalization by reducing the bias associated with the class that has the majority of the real data. This conclusion is supported by an additional study [5] which demonstrated the proposed balanced semisupervised GAN resulted in a decreased true positive rate which provides evidence that synthetic images can be used to train a more unbiased model.

However, analogous to simple data augmentation techniques, it has not been substantially demonstrated that synthetic images generated by GANs will always improve the classification of AI algorithms. Several studies have demonstrated that GAN-based data augmentation techniques have provided no improvement or negatively impacted the performance of classification models. A recent study [4] observed that there was no obvious correlation between the number of synthetic images and increased model performance when applying a progressively growing GAN with Poisson blending for road damage detection. Though the performance of the limited class was shown to improve using synthetic data from a Wasserstein GAN with gradient penalty [22], it was demonstrated that the class containing only real data was negatively impacted by the inclusion of synthetic data. The F1 score of all classes was compromised when using “non-capped” features for 1D GAN generation [26]. However, the relationship between the size of the existing real dataset and the impact on the quality of synthesized images, and the overall increase in classification accuracy has not been quantified.

To date, there have been limited studies that have investigated the performance of GANs for data augmentation within a data-compromised environment which is common within the SHM domain [3,5,8,24,25,26]. Though the application of DL techniques within SHM has been well explored and documented, the majority of these studies use large datasets (>10,000 images) [3,7,8,9,22,23,24], contrasting the real-world availability of data within SHM. Furthermore, this trend can be extended to recent studies implementing GANs for data augmentation, focusing on their effectiveness for addressing imbalanced datasets [3,5,7,8,22,25] and missing data samples [6,35,37], but these have not widely addressed GAN-based applications in data-scarce environments. Some evidence of this has been presented by Pei et al. [23] who observed that the increase in model performance with the inclusion of synthesized deep convolution GAN images decreases when an increasing amount of real training data is used. However, the amount of synthetic data added to existing data is arbitrary and there have been no studies that investigate the correlation between the amount of synthetic data and the impact on overall classification accuracy.

This paper presents an investigation into the effectiveness of a GAN architecture for the data augmentation of a limited multiclass dataset for the classification of surface damages on concrete structures. The impact of the size of the existing real dataset is quantified with respect to the diversity of images generated by the GAN structure. The diversity of the generated images as they relate to the real dataset is quantified using a centroidal-based dimensionality reduction approach. The radius and density of the clusters obtained from the proposed approach are used to correlate model performance with training sample diversity. Finally, the synthetic images are applied to a limited dataset to access the effect of generated samples on classification performance for various dataset sizes. This paper, for the first time, has explored synthetic data diversity and the impact it has on classification accuracy for limited datasets. The remainder of the paper is organized as follows. The proposed methodology, including a brief overview of the GAN, CNN, and centroidal-based dimensionality reduction approach used in this study, is provided in Section 2. Section 3 described the dataset used in this study with its implementation explained in Section 4. The results from the parametric study are summarized in Section 5 and the conclusion of the study is detailed in Section 6.

## 2. Background

This section provides an overview of the various models and techniques applied in this study including GANs, CNNs, and centroidal-based dimensionality reduction.

### 2.1. GAN for Data Augmentation

A generative model is an unsupervised machine-learning (ML) method that automatically discovers and learns the patterns in input data so that the model can generate new data samples that plausibly could have been drawn from the original dataset. A Generative Adversarial Network (GAN) is a class of generative models in which two neural networks compete to generate new, synthetic instances of data that appear to be real [32,33,34,35,35]. Adversarial processes are used to train two neural networks, generator and discriminator, that compete with each other until the desired equilibrium is reached. In this scenario, the generator network G(Z) takes random noise (Z) as input and attempts to produce data that is substantially similar to the dataset. The discriminator network D(X) on the other hand accepts generated and real data as input and attempts to distinguish between generated and real data. The output of the discriminator is the probability that the input data comes from the real dataset.

The objective function of this entire process can be expressed as follows based on the generator attempting to minimize the following function while the discriminator tries to maximize it [32]:

(1) $$\min_{G}\;\max_{D}\;L\left(D,G\right)=E_{x}\left[logD\left(x\right)\right]+E_{z}[log\left(1-D\left(G\left(z\right)\right)\right)$$

where D(x) is the discriminator’s estimate of the probability that real data instance *x* is real, $E_{x}$ is the expected value over all real data instances, G(z) is the generator’s output when given noise *z*, D(G(z)) is the discriminator’s estimate of the probability that a fake instance is real, $E_{z}$ is the expected value over all random inputs to the generator. The equilibrium point for the above function is that the generator should generate real-like data and the discriminator should output the probability of 0.5 as the generated data is almost the same as the real data, i.e., there is no way of knowing whether the new data coming from the generator is real or fake with equal probability.

The common practice for ML applications is to provide as much data as the model can take. This is because in most ML applications feeding more data enables the model to predict better. Few-Shot Learning (FSL) [39] is a field of ML for classifying new data when there are only a few training samples available for supervised learning. Consider a learning task *T*, FSL deals with a data set $D=\{D_{train},D_{test}\}$ consisting of a training set $D_{train}={\left\{\left(x_{i},y_{i}\right)\right\}}_{i=1}^{I}$ where *I* is small, and a testing set $D_{test}=\left\{x^{test}\right\}$. Let p(x,y) be the ground-truth joint probability distribution of input *x* and output *y*, and $\hat{h}$ be the optimal hypothesis from *x* to *y*. FSL learns to discover $\hat{h}$ by fitting $D_{train}$ and testing on $D_{test}$. To approximate $\hat{h}$, the FSL model determines a hypothesis space *H* of hypotheses h(·;θ), where θ denotes all the parameters used by *h*. Here, a parametric *h* is used, as a non-parametric model often requires large data sets, and thus is not suitable for FSL. An FSL algorithm is an optimization strategy that searches *H* in order to find the θ that parameterizes the best $h^{☆}\in H$. The FSL performance is measured by a loss function $l(\hat{y},y)$ defined over the prediction $\hat{y}=h\left(x;\theta \right)$ and the observed output y.

### 2.2. CNN for Damage Classification

Deep-learning algorithms, in particular, CNNs, have seen increasing use within the SHM as a mechanism to conduct an autonomous inspection of aging structures [10,11,12,13]. The benefit of using CNN-based algorithms is due in part to their autonomous feature extraction capabilities when compared to traditional ML methods such as Random Forest, Support Vector Machines, or k-means Clustering. For example, given a set of input training data that includes a binary classification between undamaged and cracked concrete surfaces, CNN algorithms are able to extract features from the images which are used to distinguish between the two classes. Therefore, a fully trained CNN would be able to classify future images of undamaged and damaged structures without direct user intervention. The autonomous nature of this process, therefore, allows inspections to be carried out in a time-efficient and more economically beneficial manner compared to traditional approaches. To extract feature maps from a given set of 3D input data that has dimensions width, height, and depth, the dot product of the input data and learned convolutional filter is taken such that an activation function can be applied to each element within the feature map [40]. Therefore, the 2D (*i,j*) location value of the *k*th feature map for a the *l*th convolutional layer, $z_{i,j,k}^{l}$ can be determined as follows [40]:

(2) $$z_{i,j,k}^{l}=w_{k}^{l^{T}}x_{i,j}^{l}+b_{k}^{l}$$

where $w_{k}^{l}$ and $b_{k}^{l}$ are the weight matrix and bias for the *k*th convolutional filter and layer, and $x_{i,j}^{l}$ is the input value at location (*i,j*) of a given convolutional layer. For multi-layer CNNs that are implemented to detect non-linear or complex features, activation functions (F(·)) can introduce non-linearity to the values associated with feature maps through the following equation [40]:

(3) $$a_{i,j,k}^{l}=F\left(z_{i,j,k}^{l}\right)$$

where $a_{i,j,k}^{l}$ is the activation value given the application of an activation function (commonly sigmoid, hyperbolic tangent, or rectified linear units are used) to a given feature value. The output of the $z_{k}^{l}$ becomes the input ($z_{k}^{l+1}$) of the l+1 layer; thus a CNN is formed by stacking convolutional layers with proceeding activation functions to create a multilayered network. Furthermore, pooling layers are often integrated after convolutional layers to reduce the number of parameters and computation time [40]. Similar to a convolutional layer, the filter is applied to the input of the layer; however, the output of the layer is based on (1) taking the maximum value contained within the filter (max pooling) or (2) taking the average of all elements within the filter (average pooling) such that [40]:

(4) $$y_{i,j,k}^{l}=\mathrm{pool}\left(a\left(z_{m,n,k}^{l}\right)\right),\forall \left(m,n\right)\in R_{ij}$$

where $R_{ij}$ is the area surrounding location (*i,j*) where the pooling operation is applied. Fully connected layers are used to flatten the inputted data into a single vector. Additionally, fully connected layers can be used to reduce the dimensionality of the vector before classification occurs. Once the final fully connected layer has been established, a Softmax layer is used to determine the probability that a single sample of the inputted data belongs to a class ‘*j*’ such that [40]:

(5) $$p_{j}=\frac{e^{z_{j}^{\left(i\right)}}}{\sum_{l=1}^{K}e^{z_{l}^{\left(i\right)}}}$$

where $p_{j}$ is the probability that the inputted data belongs to class *’j’*, $z_{j}^{\left(i\right)}$ is the activations of the fully connected layer prior to the Softmax classification, and *K* is the number of classes. Therefore, CNNs with varying architecture can be established with the capabilities of distinguishing between several classes of data making them a viable option for implementation within an SHM framework.

## 3. Proposed Framework

**Figure 1:**
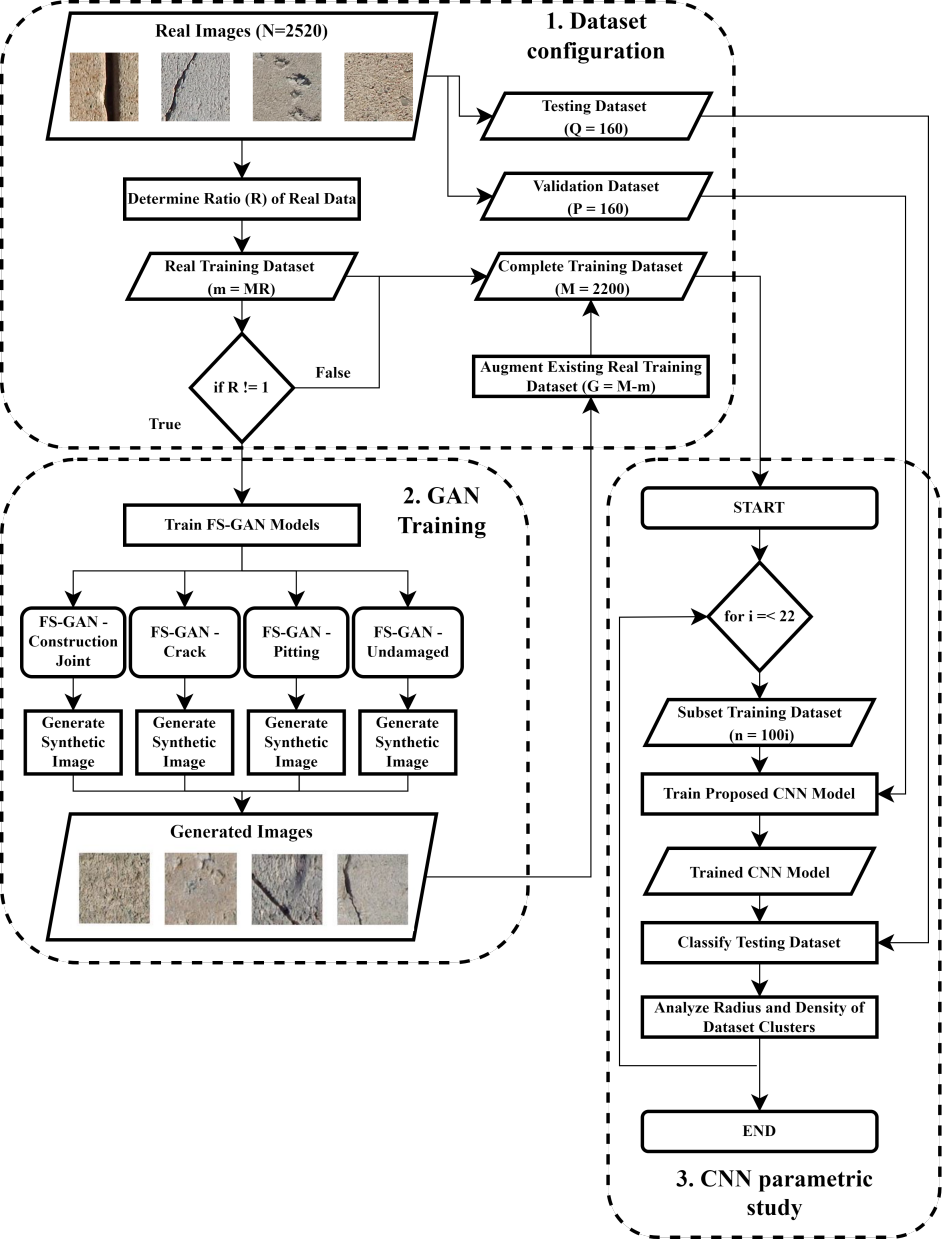
Proposed framework for the data augmentation of multiclass datasets using GANs for CNN classification.

The proposed framework for the study of multiclass GAN to generate synthetic images with limited data is depicted in Figure 1. The evaluation of the performance of augmented datasets is divided into three subroutines within the model pipeline, (1) dataset configuration, (2) GAN training, and (3) CNN parametric study. Through the study of synthetic data generation using limited data for data augmentation, the following relationships will be established:

(1) The effect of the number of samples used to train the GAN network and the overall diversity of the synthetic images generated will be characterized. Existing GAN networks for data augmentation in SHM have not quantified the effect of limited data on synthetic image diversity. Though the relative quality of synthetic images generated by GANs is often high, if the produced images are too similar to one another then they would not contribute to increasing model generalization when training a CNN.
(2) The effectiveness of data augmentation techniques is based on the size of the existing training dataset. It has been well established that for imbalanced datasets the addition of synthetic samples through data augmentation greatly improves the performance of the model [3,5,7,8]. However, studies [4,22] have shown that when the number of real training samples is sufficiently great and the classes are relatively balanced, the addition of synthetic data provides only a minimal increase in classification performance. Therefore, the relative performance increase with respect to a number of existing real training samples will be quantified for multiclass GAN-based data augmentation.

### 3.1. Dataset Configuration

Digital photography is a common mode of data acquisition within the SHM domain [1,2,3,4,5,7,8,9,10,11,12,13,14,15,16,17,20,21,22,23,24,25,26,27,28,29,41,42] as cameras can be readily mounted to unmanned land and aerial vehicles which are able to access damaged structures that have limited accessibility for inspectors [2]. This study could be further extended to video as well because videos can be considered a series of frames (images) that are viewed in a sequential order. Therefore the proposed framework could be applied to each frame of the video sequence as if each frame of the video is an independent image. Given a database that contains a total of *N* real image samples over four equally distributed classes (construction joint, crack, pitting, and undamaged), a significant selection of the total dataset is chosen to represent the training dataset in the analysis. The *N* images are partitioned into training, validation, and testing datasets based on a 80%/10%/10% split. Therefore, the training dataset is comprised of *M* images where M = 0.8 N which in this study equates to 2200 images. Similarly, the validation (*P*) and testing (*Q*) datasets equate to 160 images each; however, these datasets are used to validate the trained CNN architecture. The equal distribution of classes that are represented in the total dataset of size *N* is also represented in the training, validation, and testing datasets to ensure that class imbalance does not impact the results of the study. However, since this study explores data augmentation for a limited dataset, a variable fraction of real image data is chosen to represent the ‘Real Training Dataset’. The amount of training samples selected is determined based on a predefined ratio *R* which has a range of 0<R≤1 where *R* = 1 would represent the entire training dataset with *M* samples. The ’Complete Training Dataset’ of size *M* is implemented to determine the baseline performance of the model on a dataset that can be considered sufficiently large with respect to adequately training the proposed CNN network. As such, when *R* is equal to 1, no data augmentation occurs and the existing training data are used directly to train the proposed CNN network.

Those *R* values not equal to 1 represent real training datasets that contain *m* samples where m≤M represents a small subset of all the available training data for analysis. For those *R* not equal to 1, the ’Real Training Dataset’ would contain *m* = *MR* samples and these are considered ‘limited datasets’ which theoretically would result in a trained CNN with reduced performance due to the limited training data being available to the network. As such, for those datasets with *R* < 1.00, the *m* samples within the ‘Real Training Dataset’ would be used to train the FS-GAN implemented in this analysis to generate new samples such that the existing ‘Real Training Dataset’ could be augmented using synthetic images. Furthermore, the range of *R* is exclusive of 0 because, to train the FS-GAN model, there must be real data to train the generator model to learn the characteristics of the classes within the dataset. Once the ‘Real Training Dataset’ has been used to train the FS-GAN models, the generator which represents each class can be used to generate images that augment the existing datasets. For "Real Training Datasets" with size m the number of samples that are generated by the FS-GANs is equivalent to *G = M - m* and these are similarly equally distributed across all four classes. These samples are used to augment the existing ‘Real Training Images’ which results in a ‘Complete Training Dataset’ of size *M* containing both real and synthetic images.

### 3.2. GAN Training

This study focuses on the generation of synthetic samples in low data environments, thus the selected GAN architecture must be capable of generating samples that have high visual quality while using a small number of samples to train the network. It is well established that though GANs possess the capabilities to generate new image samples, the training of these networks requires a significant number of images to accurately train the generator network. As such, the model implemented in the proposed research is from a study conducted in 2021 by Liu et al. [43] in which a hybrid Few-Shot GAN (FS-GAN) was created for the generation of high-fidelity images. The model proposed by Liu et al., is capable of generating high-resolution images while only being trained on less than 100 samples, making it ideal for implementation in the current study. The authors improved upon the traditional GAN architecture by introducing skip-layer excitation modules and a multi-resolution encoder–decoder structure for the discriminator to reduce computational complexity during training while maintaining high resolution in the generated samples. More details about the discriminator and generator networks can be found in the original [43].

An FS-GAN model is trained for each of the classes. The objective of this process is to establish a trained generator that is capable of generating synthetic images for each of the classes belonging to multiclass damage detection. Given a ‘Real Training Dataset’ of size *m* which is equally distributed amongst all classes, the individual classes are separated from each other and become an individual dataset for the training of the FS-GAN model. For example, in this study, there are four classes—crack, construction joint, pitting, and undamaged; each of these classes would have a homogeneous dataset that is comprised of images belonging to only that particular class. The reasoning behind separating the ‘Real Training Dataset’ and training a separate model for each class during the FS-GAN training is to ensure that the generator can properly generate the features of a particular class rather than generating features that may be a blend of all four classes. Using each dataset belonging to their classes, an FS-GAN model is trained such that a trained generator is established for each classification. The trained generator can subsequently be used to generate *G* synthetic images which are used to augment the existing ‘Real Training Dataset’ to establish a ‘Complete Training Dataset’ which consists of both real and synthetic images.

### 3.3. CNN Parametric Study

A parametric study is conducted by which the CNN model is trained with progressively increasing amounts of data each iteration. The number of iterations is relative to the number of additional training samples that must be added to each iteration originating from the ‘Complete Training Dataset’. The total number of images within the ‘Complete Training Dataset’ in this study is 2200 images and 100 images (25 images per class) per iteration are selected to comprise the ‘Subset Training Dataset’ such that there are enough data points (22 points) to observe a discernible trend in the data. Therefore, the CNN parametric study consisted of 22 iterations in which 100 new training samples are added to the ‘Subset Training Dataset’ to train the proposed CNN model. For ’Complete Training Datasets’ which are comprised of both real and synthetic image samples, the real image samples are added first to the ‘Subset Training Dataset’ followed by the synthetic images. This is to ensure that the effect of synthetic data on CNN classification can be quantified. If the training data are randomized between real and synthetic samples, it would not be possible to correlate the trend of the CNN performance to a particular data type. For example, if the ‘Complete Training Dataset’ has an *R* = 0.5, the first 11 iterations would have 100 real images added each iteration to the ‘Subset Training Dataset’ followed by the remaining 11 iterations which would add 100 synthetic images instead. The ’Subset Training Dataset’ is established, and it is used to train the proposed CNN network. During training, the validation dataset is used to assess the performance of the CNN model, allowing for hyperparameter tuning of variables to prevent overfitting on the training dataset. Once the proposed CNN model is trained, the trained model is applied to a testing dataset to determine the performance of the fully trained model on an unseen dataset.

### 3.4. Cluster and Density Analysis of Datasets

The diversity of the training dataset containing real and synthetic samples is quantified by clustering the ‘Subset Training Dataset’, validation, and testing datasets using a centroidal-based approach. The quality and diversity of synthetics samples generated by GAN architectures are important metrics to consider with respect to CNN-based classification performance. Low-quality synthetic images, when used for training, often reduce the classification performance of CNN networks as the features have a reduced detail which is more difficult to classify by the trained model. Issues with poor model generalization are often experienced too when using a training dataset with a low diversity of samples. Since the features of the dataset are similar, CNNs tend to fit the model parameters to the noisy features of the data rather than optimize based on a general understanding of the features as they relate to the definition of the classes. The result is a CNN model that performs well on the training data but has reduced performance when classifying unseen testing datasets. In SHM, models with poor generalization are detrimental to the inspection process as structural damages, such as cracks, may have a wide range of features associated with the classification and therefore perform poorly for the damage classification task. As a result, engineers may be required to retrain existing models to include additional training samples which delay the inspection process resulting in economic and social losses in the affected areas.

**Figure 2:**
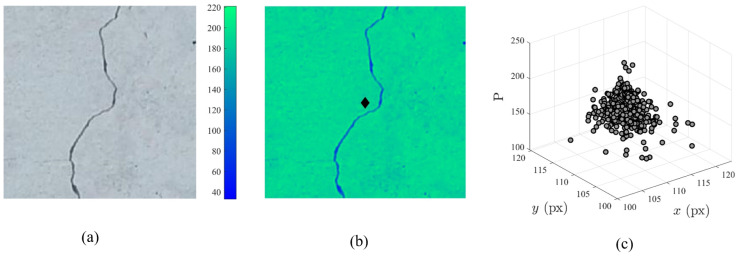
(**a**) Sample training image of concrete crack, (**b**) centroid location (black) based on pixel intensity, and (**c**) centroid location of all images within the ‘crack’ training dataset.

However, it is intrinsically difficult to observe the diversity of samples through clustering algorithms with respect to image-based datasets due to the high-dimensional nature of RGB images. Therefore, a centroid-based dimensionality reduction inspired by the work of Barbosh et al., 2022 [44] is used to quantify the diversity of images within a given dataset classification as depicted in Figure 2. The inspiration for this technique is derived from the geometric process by which a centroid of a composite shape is determined. The centroid of a composite shape can be considered the weighted sum of the centroids of all shapes where the weights given to each centroid are based on the area associated with each centroid. Similarly, an image can be considered a composite shape where the centroid of each pixel can be determined and the weight of each pixel can be represented by its pixel intensity. Given an RGB image as depicted in Figure 2a, the number of channels is reduced by converting the RGB image to a greyscale image. As RGB images consist of a 3D matrix of size (*M,N,c*) where *c* is a channel associated with the red, blue, and green spectrum of the image, the image can be converted to grayscale [44] through Equation (6):

(6) $$P_{mn}=0.2989P_{R_{mn}}+0.5870P_{G_{mn}}+0.1140P_{B_{mn}}$$

where *m* is the integer value representing the location of the pixel along the length of the image, *n* is the integer value representing the location of the pixel along the width of the image, $P_{R_{mn}}$ is the pixel intensity of the red channel at pixel location (*m,n*), $P_{G_{mn}}$ is the pixel intensity of the green channel at pixel location (*m,n*), $P_{B_{mn}}$ is the pixel intensity of the blue channel at pixel location (*m,n*), and $P_{mn}$ is the pixel intensity of the grayscale image at pixel location (*m,n*). Once the RGB image has been converted to grayscale, the centroid of the image can be determined as shown in Figure 2b based on the pixel intensity of the image as represented by the color bar. The centroid of the grayscale image can be considered as the ‘moment’ of the image, or the point at which all pixel intensities ($P_{mn}$) at distance (*x,y*) have been balanced. Therefore, the dimensionality of the 2D grayscale image is further reduced to a 3D point which can be correlated to others within the dataset. The coordinates of the 3D point ($x_{c},y_{c},P_{c}$) can be determined using Equations (7)–(9) based on the characteristics of the grayscale image as described below:

(7) $$x_{c}=\frac{\sum_{n=1}^{N}\sum_{m=1}^{M}x_{mn}P_{mn}}{\sum_{n=1}^{N}\sum_{m=1}^{M}P_{mn}}$$

(8) $$y_{c}=\frac{\sum_{n=1}^{N}\sum_{m=1}^{M}y_{mn}P_{mn}}{\sum_{n=1}^{N}\sum_{m=1}^{M}P_{mn}}$$

(9) $$P_{c}=\frac{\sum_{n=1}^{N}\sum_{m=1}^{M}P_{mn}}{MN}$$

where $x_{c}$ is the location of the centroid along the length of the image, $y_{c}$ is the location of the centroid along the width of the image, $P_{c}$ is the average pixel intensity across the entire image at the centroid, $x_{mn}$ is the centroid of the pixel located at (*m,n*), $y_{mn}$ is the centroid of the pixel located at (*m,n*), *M* is the total number of pixels along the length of the image, and *N* is the total number of pixels along the length of the image. Assuming each pixel of the image has a unit length and width, the centroid of any pixel at location (*m,n*) can be considered the following ($x_{c},y_{c}$) = (m−0.5,n−0.5); for instance, the centroid of the pixel located at (1,1) would be (0.5,0.5). This process is repeated for all images within the dataset and a 3D cluster representing the centroids of all images with the dataset for a particular class (’crack’) can be observed as depicted in Figure 2c. To quantify the diversity of the images within the dataset, the density of the cluster is calculated assuming the volume that encompasses the 3D points is a perfect sphere. Given a number of 3D points equal to the number of images within a particular class, the centroid of all points can be determined from Equations (10)–(12):

(10) $$x_{cl}=\frac{\sum_{i=1}^{I_{p}}x_{ci}}{I_{p}}$$

(11) $$y_{cl}=\frac{\sum_{i=1}^{I_{p}}y_{ci}}{I_{p}}$$

(12) $$P_{cl}=\frac{\sum_{i=1}^{I_{p}}P_{ci}}{I_{p}}$$

where ($I_{p}$) is the number of points represented in the cluster. Once the centroid of the cluster has been determined, the 3D Euclidean distance for each point within the cluster to the cluster center (($x_{c1},y_{c1},P_{c1}$),($x_{c2},y_{c2},P_{c2}$)…($x_{cI_{p}},y_{cI_{p}},P_{cI_{p}}$)) is determined using Equation (13):

(13) $$d_{i}=\sqrt{{\left(x_{ci}-x_{cl}\right)}^{2}+{\left(y_{ci}-y_{cl}\right)}^{2}+{\left(P_{ci}-P_{cl}\right)}^{2}}$$

where $d_{i}$ is the distance between the centroid of the entire cluster and the *i*th point within the cluster. The radius of the sphere containing the cluster is therefore taken as the maximum of all $d_{i}$ when calculating the volume of the cluster. Finally, the density of the cluster can be determined from the following equation:

(14) $$D=\frac{I_{p}}{\frac{4}{3}\pi d_{max}^{3}}$$

where D is the density of the cluster in ${\mathrm{points}/\mathrm{pixels}}^{3}$, $\frac{4}{3}\pi d_{max}^{3}$ is the volume of the cluster, and $d_{max}$ is the maximum distance between the centroid of the entire cluster and a singular point contained within the cluster.

## 4. Description of Database

**Figure 3:**
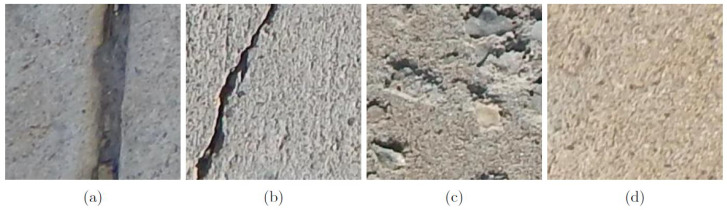
Classes of concrete surface defects used for GAN-based Data Augmentation: (**a**) construction joint, (**b**) cracks, (**c**) pitting, and (**d**) undamaged.

There exist very few benchmark datasets for training new AI algorithms in SHM. One of the most popular datasets for surface-based concrete defects of structural elements, SDNET2018 [45] is a publicly available dataset that has been created by researchers to investigate the application of AI methods for SHM. More than 56,000 segmented images of damaged and undamaged unreinforced concrete pavement and reinforced concrete walls and bridge decks are included within this dataset. A diverse collection of defects are represented by these images including cracks of varying widths, shadows, stains, voids, surface scaling, and vegetation. A 16-MP Nikon camera at a working distance of 500 mm was used to capture 4068 × 3456 px images that were further segmented into subimages [45]. Each segmented image is a 3D-RGB image with a size of 256 pixels in length by 256 pixels in width. Further details about the data acquisition can be found in the following study [45]. Figure 3a–d depicts the classes that are used in this study including undamaged, cracked, pitted, and concrete surfaces with construction joints. Furthermore, each selected image is resized to 224 pixels in length by 224 pixels in width to fit the desired input size of the proposed CNN model.

Two factors that could impact the performance of the method proposed are considered while choosing images for the creation of the dataset used in the experimentation. First, due to the low probability of observing a damaged concrete surface relative to a normal concrete surface, the number of annotated images belonging to the ‘undamaged’ class is far greater than the remaining damage-based classes. The significant difference in the number of images for each class available for training results in a biased model in which the classifier is more likely to correctly identify an image belonging to the majority class. This can be detrimental to inspection-based methods implemented in SHM as the primary objective of this analysis is to identify those images belonging to the minority class. Furthermore, the results of metrics such as accuracy lose value when quantifying the performance of classifiers and observing imbalanced data. For instance, for a binary classification problem, if the majority class is composed of 950 images compared to the minority of 50 images, correctly identifying the majority class would result in an accuracy of 95%, which is not indicative of how the model performed classifying both the major and minor class.

Secondly, many of the images contained various environmental and mechanical noises including the presence of shadows, vegetation, and blurring of various images. Similar to class imbalance, the presence of noisy images can introduce model biases during the training of a classifier. The tendency of a model is to fit the noisy features of the images (ex. background elements) rather than the features which are representative of the class itself. This may result in model overfitting, where the model performs well on the data that it has been trained on but has poor adaptation when presented with a new dataset resulting in poor performance. As such, noise-free images are chosen for the training, validation, and testing dataset as depicted in Table 1. Each class has an equivalent number of training, validation, and testing images to address issues surrounding class imbalance. The number of training samples, (550 images) and validation, and testing samples (40 images) for each class is determined based on the size of the minority class with the lowest total images (pitting—630 images). The number of validation and testing samples for each class has been limited to 40 images in order to simulate the data scarcity issues which are prevalent in SHM. For those classes with more available images (i.e., crack, construction joint, and undamaged), a sample equal to the number of images comprising the pitting dataset is determined based on random integer selection. The validation and testing datasets consisting of 40 images per class are kept separated from the model during training to ensure these datasets remain unseen. As these datasets are not involved in the training process, the testing and training dataset can be used to determine the generalization capabilities of the model on unseen datasets as well as tuning the parameters of the model during the optimization step.

## 5. Evaluation

The primary objective of this research is to study the influence of limited datasets on synthetic sample generation quality using a GAN architecture and the impact of these synthetic images on multiclass damage detection. Therefore, to explore the correlations between limited datasets and these factors, datasets of varying sizes must be implemented in the model pipeline.

### 5.1. Experiment Setup

To accomplish this, five distinct training scenarios as summarized in Table 2 are created by reducing the number of samples available in the training dataset for both the GAN and CNN models. A description of each training scenario has been provided below:

1. For Scenario 1, the training dataset is comprised of only real data. As demonstrated in Table 1, each class has 550 training samples that are extracted from the SDNET 2018 database. To create a baseline for this study, the training database (*M* = 2200) is used to train only the CNN; no generated images are used in this study.
2. For Scenario 2, the training dataset is comprised of only 415 real images for each class resulting in a real training dataset of 1660 images. For each class, the 415 images are used to train the aforementioned GAN architecture resulting in a generator for each class in the multiclass dataset. Each generator model is used to generate 135 synthetic images resulting in a total training database with a similar size as Scenario 1 but with 25% synthetic data. The combined real and synthetic dataset is then used to train the CNN architecture for multiclass damage identification.
3. For Scenario 3, the training dataset is comprised of only 275 real images for each class resulting in a real training dataset of 1100 images. For each class, the 275 images are used to train the aforementioned GAN architecture resulting in a generator for each class in the multiclass dataset. Each generator model is used to generate 275 synthetic images resulting in a total training database with a similar size as Scenario 1 but with 50% synthetic data. The combined real and synthetic dataset is then used to train the CNN architecture for multiclass damage identification.
4. For Scenario 4, the training dataset is comprised of only 135 real images for each class resulting in a real training dataset of 540 images. For each class, the 135 images are used to train the aforementioned GAN architecture resulting in a generator for each class in the multiclass dataset. Each generator model is used to generate 415 synthetic images resulting in a total training database with a similar size as Scenario 1 but with 75% synthetic data. The combined real and synthetic dataset is then used to train the CNN architecture for multiclass damage identification.
5. For Scenario 5, the training dataset is comprised of only 50 real images for each class resulting in a real training dataset of 200 images. For each class, the 50 images are used to train the aforementioned GAN architecture resulting in a generator for each class in the multiclass dataset. Each generator model is used to generate 500 synthetic images resulting in a total training database with a similar size as Scenario 1 but with 90% synthetic data. The combined real and synthetic dataset is then used to train the CNN architecture for multiclass damage identification.

Both the validation and testing datasets are the same for Scenarios 1–5 and are not introduced to the network during the training of the CNN to ensure the network is capable of generalizing with respect to the classification of structural damages. The real training data are used to train the GAN architecture, with a separate GAN model trained for each of the four classes. Once the generator model for each class is trained, they are used to generate synthetic images to augment the existing training database such that the total number of images remains 2200. Finally, the training dataset for each scenario containing both synthetic and real samples is used to train the CNN network as described and the diversity of the training samples is quantified. The validation and testing dataset size and composition are kept constant across all scenarios as the purpose of this study is to characterize the performance of the proposed CNN network on unseen datasets (such as validation and testing) when using synthetic data to train the network. Therefore, only the fact that the proposed trained CNN has not analyzed these datasets during the training process is relevant.

### 5.2. Synethic Sample Generation Using GAN

Once the database is assembled, real samples for each class are selected and used to train the FS-GAN as outlined in Table 2. A separate FS-GAN has to be trained for each class as introducing dissimilar images from other classes during training would only diminish the capabilities of the generator to synthesize new images belonging to the class. The amount of training data used for each class is equal; therefore, each class is represented by 25% of the real training data. Each FS-GAN is trained using an Adam solver for 50,000 epochs with a batch size of 8, a learning rate of 0.002, and $\beta_{1}$ and $\beta_{2}$ values equal to 0.5 and 0.999. The hyperparameters are optimized [43], and all FS-GAN training is conducted through the Python ‘Pytorch’ library using LENOVO MT 30BA ThinkStation with NVIDIA Quadro 95000 GPU and 32GB RAM and took approximately 16.5 h to completely train each FS-GAN model.

**Figure 4:**
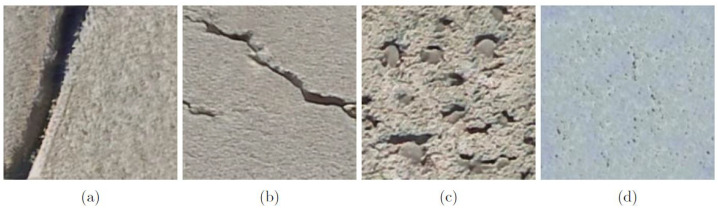
Synthetic images produced by the generator of the FS-GAN: (**a**) construction joint, (**b**) cracks, (**c**) pitting, and (**d**) undamaged.

Once training is complete, the trained model state is saved for the generator such that new images could be synthesized using the fully trained model as shown in Figure 4a–d. Each trained model is loaded and used to generate several 512 × 512 RGB images based on the number of synthetic images required for each scenario as described in Table 2. To prevent a decrease in CNN classification due to class imbalance, each of the four classes within the synthetic dataset is equally represented such that each class represents 25% of the total synthetic images generated for each class. Furthermore, as the input size for the CNN described in Section 5.2 is 224 × 224 × 3, each synthetic image as shown in Figure 4a–d is reduced in size to 224 × 224 × 3 px to ensure all images inputted into the CNN are of the same size. Once all images are generated and resized, they are added to the existing real samples to train the CNN, thus augmenting the available data for multiclass surface damage detection of concrete structures.

### 5.3. Multiclass Damage Detection Using CNN

Proceeding the augmentation of the existing dataset, the combined real and synthetic training data are used to train a CNN for multiclass damage detection of concrete structures. The process of training the CNN is iterative as demonstrated in Table 3, with an increasing amount of training data added each iteration such that the classification performance can be correlated to the amount of synthetic/training data that is used. For each iteration, 25 new training samples for each class of data are added to the existing dataset, increasing the available amount of data for the network during the training process. The training database size is increased using only real images until a point where there are no longer any real images to be added to the dataset at which point synthetic samples are used to further augment the training dataset. The systematic order in which samples are added to the training dataset is done such that there is a discernible point at which synthetic images are added during training such that the overall impact on classification performance can be quantified. Once the training dataset has been established for each iteration, the proposed CNN is initialized using randomized weights and biases and the training process begins.

**Figure 5:**
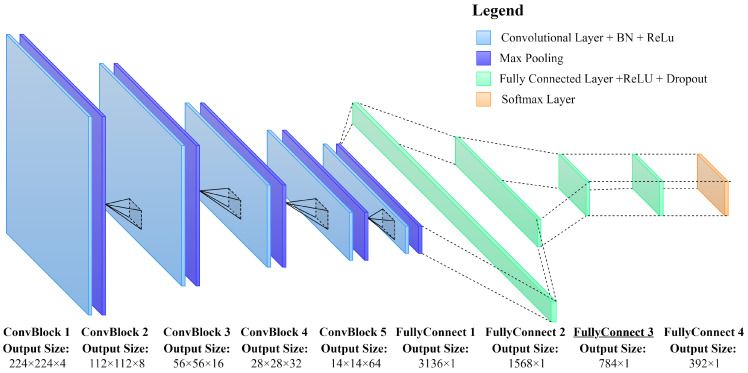
Configuration of the proposed CNN model.

For this study, a simple CNN architecture is developed as shown in Figure 5. The number of layers and parameters of the layers are determined through a random search which resulted in the best performance on the validation dataset given a training dataset consisting of only real training data. The proposed network consists of five convolutional blocks, each one with two layers, (1) a convolutional layer and (2) a max pooling layer. Parameters for each convolutional layer are consistent across all blocks with a kernel size of 2 × 2, a stride length of 1, and padding of [1,1]. The depth of each convolutional layer increased by a factor of 2 for each convolutional block such that the depth of the filters increased from 4 to 64 across the five convolutional blocks. A max pooling layer is implemented at the end of each convolutional block to reduce the size of the filters by half such that the initial image size of 224 × 224 × 3 is reduced to 7 × 7 × 64 across all convolutional blocks. After the convolutional process, the resulting output is flattened into a fully connected layer with an initial vector size of 7 × 7 × 64 = 3136. Three additional fully connected layers are used to reduce the length of the flattened vector from [3136,1] to [392,1] with each layer reducing the length by a factor of 2. Finally, the output of the final fully connected layer is reduced to a size of [4,1] based on the number of classes under investigation and a softmax probability layer is applied to determine the probability of the inputted image belonging to a particular class within the data. In total, the number of trainable parameters for the proposed network is approximately equal to 6.5 million.

**Figure 6:**
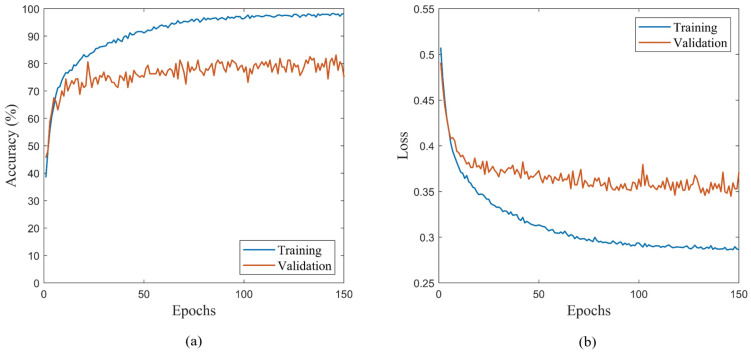
Training and validation (**a**) accuracy and (**b**) loss for the proposed CNN.

Once the configuration of the proposed CNN is established, the architecture is trained in iterations based on the details provided in Table 3 for each scenario. The network is trained using an Adam solver for 150 epochs with a batch size of 8, a learning rate of 0.0001, and $\beta_{1}$ and $\beta_{2}$ values equal to 0.9 and 0.999. The training parameters are set to these values for all iterations to ensure that any change in network performance is a result of the training dataset composition. Furthermore, since the weights and biases of the CNN are initialized from a random seed at the start of network training, this can introduce variability in the weights and biases of the final trained network. As such, the random seed is fixed to a certain value such that the weights and biases are initialized from the same value each time. The value of the random seed is chosen based on a parametric study by which 10 versions of the proposed CNN model are trained using different seeds. The seed that produced the best performance based on the proposed indicators is used as the fixed seed value for the study. The training and validation accuracy and loss are plotted during the training process as demonstrated by Figure 6a,b to determine the number of epochs required in addition to observing the level of overfitting the network experienced. In this example, the trained network depicted in Figure 6a,b shows some level of overfitting on the training data as the difference in accuracy and loss plots for the training and validation datasets is relatively high. The network, therefore, fits the learned parameters on the distinct features of the training dataset as opposed to generalizing the features and thus has reduced performance on the unseen validation dataset.

**Figure 7:**
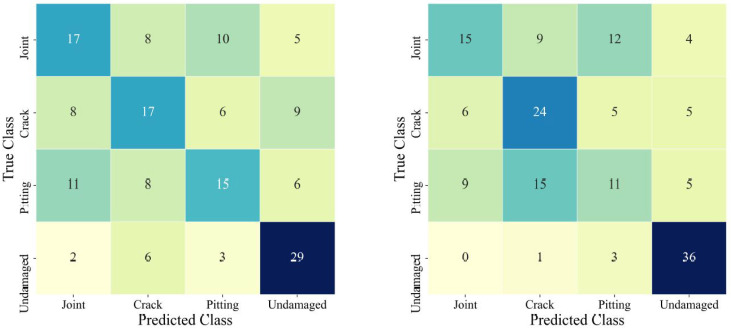
Confusion matrices for (**left**) validation dataset and (**right**) testing dataset for Scenario 1 using 100 real training samples.

Once the proposed CNN has been fully trained, confusion matrices are generated for the validation and testing datasets as depicted in Figure 7a,b to determine the performance of the network. In this study, accuracy, precision, recall, and F1 score are calculated and used to evaluate the overall performance of the network for each iteration and scenario. Given a confusion matrix, the accuracy, which describes the level of correctness the network achieves regarding a given classification task, can be determined from the following equation:

(15) $$\mathrm{ACC}=\frac{\mathrm{TP}\;+\;\mathrm{TN}}{\mathrm{TP}+\mathrm{FP}+\mathrm{TN}+\mathrm{FN}}$$

where TP is the true positive value representing the number of instances where the network correctly identifies samples belonging to the positive class, TN is the true negative value representing the number of instances where the network correctly identifies samples belonging to the negative classes, FP is the false positive value representing the number of instances where the network incorrectly identifies samples belonging to the positive class and FN is the false negative value representing the number of instances where the network incorrectly identifies samples belonging to the negative class. For instance, given ‘Joint’ as the positive class in Figure 7a, and given the position of each value in the confusion matrix can be represented by a pair of numbers ([row,column]), the elements belonging to each variable would be the following: TP = [1,1], TN = [2:4,2:4], FP = [2:4,1], and FN = [1,2:4]. Similarly, TP, TN, FP, and FN can be used to determine the precision, recall, and F1 scores from the confusion matrices. Precision is a ratio indicating how many true positive samples are correctly identified by the network in comparison to false positives. Recall is a ratio indicating how many true positive samples are correctly identified out of all possible true positive samples analyzed by the network. F1 score is a metric that takes the harmonic mean of both precision and recall scores and represents it as a singular value. Equations (16)–(18) demonstrate how precision, recall, and F1 score are calculated:

(16) $$\mathrm{Precision}=\frac{\mathrm{TP}}{\mathrm{TP}+\mathrm{FP}}$$

(17) $$\mathrm{Recall}=\frac{\mathrm{TP}}{\mathrm{TP}+\mathrm{FN}}$$

(18) $$F1\;\mathrm{Score}=\frac{2\mathrm{TP}}{2\mathrm{TP}+\mathrm{FP}+\mathrm{FN}}$$

To get a representative value for each performance indicator across the entire dataset, the results of each class for accuracy, precision, recall, and F1 score are averaged across all classes. Therefore, Mean Accuracy (mACC), Mean Precision (mP), Mean Recall (mR), and Mean F1 score (mF1) represent the average performance with respect to each performance indicator across all classes for a given dataset. For example, given Figure 7a,b and Equations (16)–(18) the mACC, mP, mR, and mF1 would be 74.38%, 0.49, 0.48, 0.48 and 76.88%, 0.54, 0.52, 0.52 for the validation and testing datasets, respectively.

### 5.4. Centroidal Analysis of Images for Diversity Quantification

**Figure 8:**
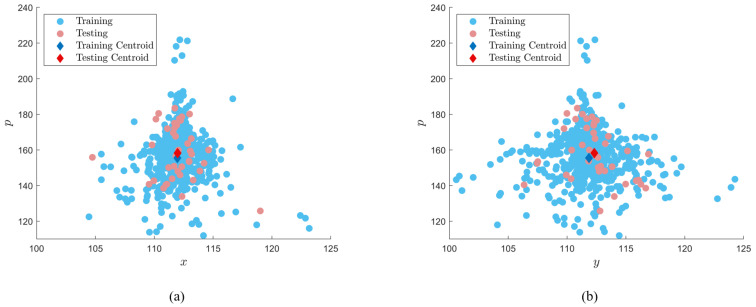
Clustering of training and testing datasets along (**a**) x-direction and (**b**) y-direction for the class ‘Crack’.

Following the performance evaluation of the network as outlined in Section 5.2, the training dataset can be converted into a 3D point cloud based on the process outlined in Section 3. For example, Figure 8a,b presents the clustering of the real training and testing images belonging to the ‘crack’ class along the x- and y-directions of the cluster. From Figure 8, the overall dispersion of samples along the x- and y-directions are relatively high for training, validation, and testing datasets concluding that these datasets have a diverse representation of the given class. As the validation and testing datasets do not change across the proposed scenario and iteration framework, the validation and testing clusters for each class remain the same throughout the study. However, the training dataset cluster varies greatly with the number of training images implemented in addition to whether those images belong to ‘real’ or ‘synthetic’ sub-classes of data. Therefore, the density and radius of the training cluster are monitored across each scenario and iteration using Equations (13) and (14) to determine the variation of clusters with synthetic image ratio and dataset size. Furthermore, the centroid of the training cluster is compared to the centroid of both validation and testing datasets and the distance between the centroid of each cluster is calculated using Equation (13) where the x, y, and *p* values represent the centroid of the training, validation, and testing cluster, respectively.

### 5.5. Results of the Parametric Study

**Figure 9:**
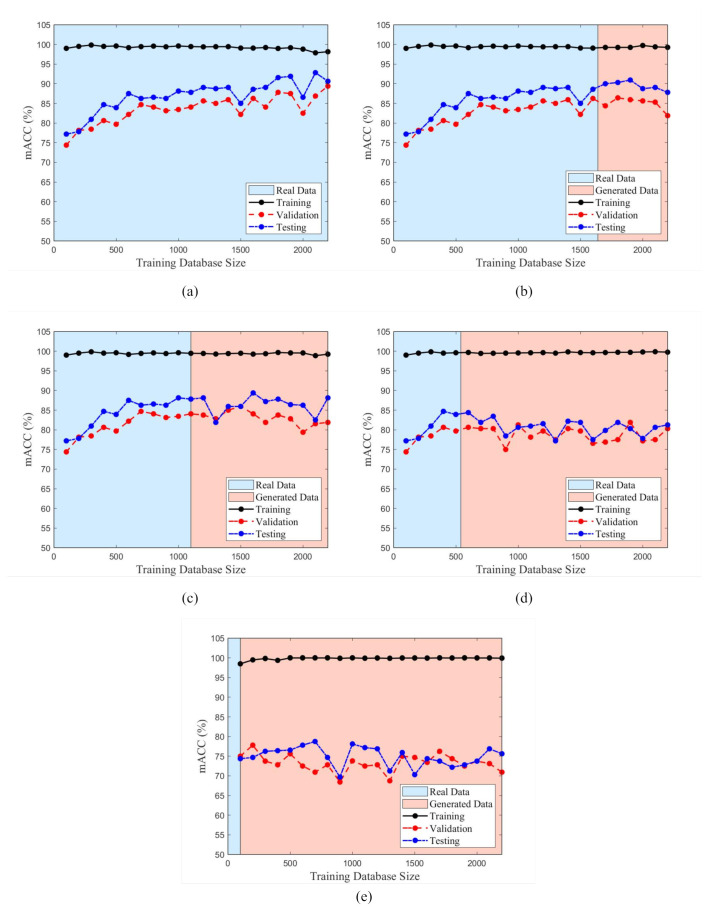
mACC of proposed CNN using different synthetic image ratios: (**a**) Scenario 1—0%, (**b**) Scenario 2—5%, (**c**) Scenario 3—50%, (**d**) Scenario 4—75%, and (**e**) Scenario 5—90%.

Figure 9a,b quantifies the variation of mACC with respect to increasing training database size for different synthetic image ratios present within the dataset. The typical performance of a CNN is demonstrated in Figure 9a, where it is observed that adding additional training samples improves the overall performance of the network. At low training dataset sizes (i.e., *n* = 100), though the trained model performs well on the training dataset, it experiences a significant decrease in performance of −24.62% and −21.81% as summarized in Table 4 for the validation and testing datasets. This disparity in performance between training and validation/testing datasets is a result of overfitting the learned parameters on the training dataset resulting in a model that has poor generalization and is unable to accurately classify images the model has not observed previously which are represented in the validation and testing dataset. As the amount of training data is increased in Figure 9a it can be observed that the severity of overfitting decreases as the generalization of the model improves as new samples are introduced in training. Furthermore, it is observed that there are diminishing performance increases as the dataset becomes significantly large suggesting that the model becomes saturated during the learning process as more and more training samples are provided. Though this model continues to experience some level of overfitting, the amount is greatly reduced at large training dataset sizes (i.e., *n* = 2200) with a decrease in performance of −8.79% and −7.51% as observed in Table 4.

However, those scenarios in which synthetic training data is used as represented by Figure 9b–e contrast with the expected performance of the proposed CNN. From Table 4, it can be observed for Scenarios 2–5 that the training accuracy remains relatively similar to Scenario 1 which involved training the CNN using entirely real training samples. However, the performance on validation and testing datasets is dramatically reduced. For Scenario 2 (Figure 9e), which uses 90% synthetic data for training, the performance on the validation and testing dataset using a large number of training samples (*n* = 2200) is worse when compared to the performance using limited training samples (*n* = 100). Moreover, for Scenarios 2–4 (Figure 9b–e, it can be observed that once synthetic data is used to supplement the existing real training dataset, the classification accuracies of validation and testing datasets no longer increase and begin to oscillate around a particular level of accuracy. Comparing the performance of the trained model on validation and testing data using only real data (red section of the graph) for the performance of the trained model using synthetic and real data (blue section of the graph) indicates that the model accuracy decreases for all cases as summarized in Table 5. It can be observed that there is an average decrease of model mACC of −1.6% with a range between −0.8% and −3.2%. In Scenario 5, which used the least synthetic data, it is observed that the model performance increased by a small margin of 0.9%. However the remaining cases observed all demonstrated decreased performance at classifying datasets with the use of the synthetic training samples.

The relative size of the testing dataset that is analyzed by the proposed CNN model must be considered as biases may be introduced when calculating performance indicators for small datasets. For example, for the testing dataset size implemented in this study (160 images), the negative impact a single image has on the accuracy if that image is a FP or FN value is 1/160 = 0.625%. Therefore, the smaller the dataset, the more influence proportionally each individual image has on the performance metrics of the network. Therefore, to further examine the impact of the synthetic dataset while decreasing the impact of each individual image on a given parameter can be considered negligible as it relates to the entire testing dataset if the dataset is large. As such, Table 6 demonstrates the effect of testing dataset size at it relates to classification accuracy for Scenario 5, but with much larger testing datasets. It can be observed that increasing the testing dataset size resulted in a decreased accuracy for the majority of training configurations with 30% of iterations showing an increase in classification accuracy. However, the relative increase/decrease in classification accuracy is marginal when comparing the standard deviation (SD) and range for each training dataset size. The majority of cases have low SD ($SD_{average}$ = 1.23%), which demonstrates that the majority of values fall within 1–2% of the average of all testing dataset sizes, demonstrating that the testing dataset size has minimal effect even when increased to a sufficiently large dataset where each sample would have a marginal impact on the accuracy (1/2160 = 0.05%). This is further evident when comparing the average range between minimum and maximum accuracy values of all training dataset sizes for testing dataset classification (Average Range = 3.5%). Though there are some training dataset sizes which demonstrate increased variability (i.e., Q = 700, $SD_{average}$ = 2.58%, Average Range = 7.40%), overall the testing accuracy remains only marginally affected by the training dataset size, in addition to the testing dataset size.

**Figure 10:**
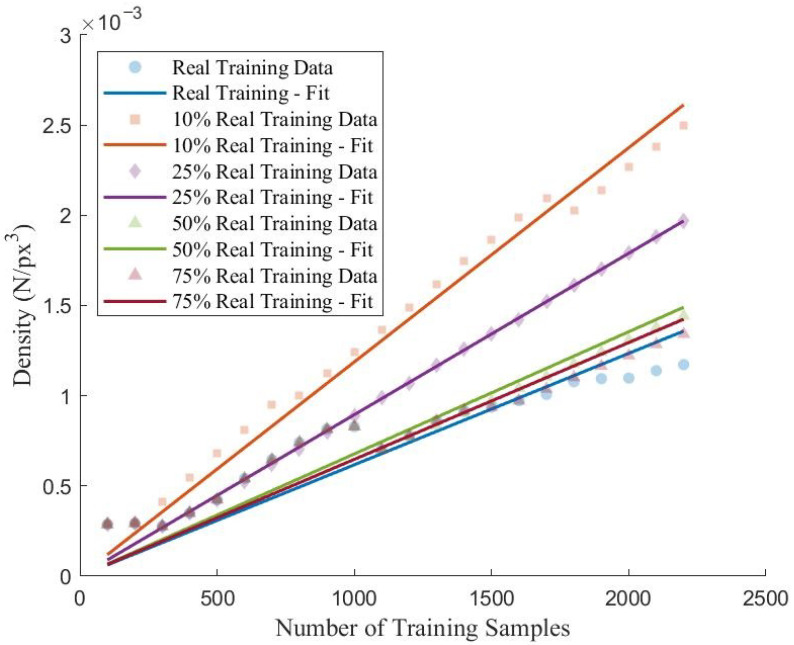
Training dataset cluster density for various synthetic image ratios for all classes.

**Figure 11:**
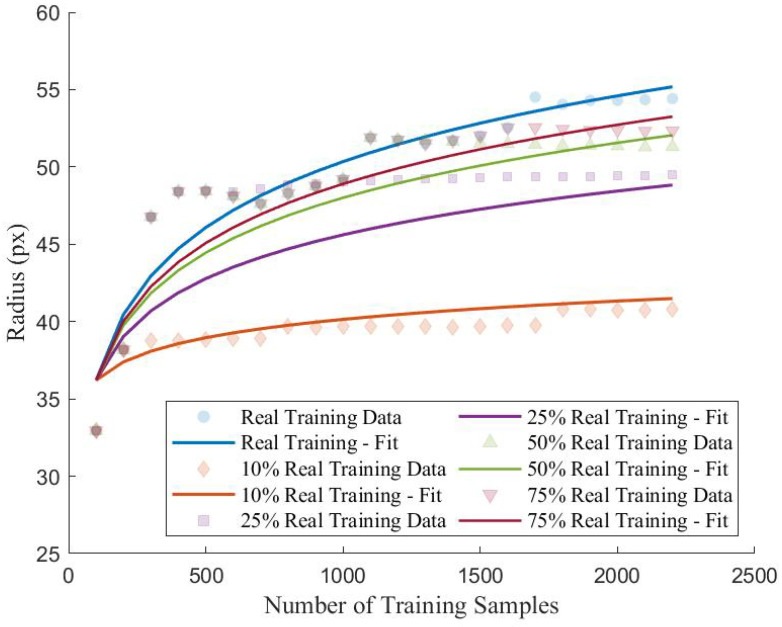
Training dataset cluster radius for various synthetic image ratios for all classes.

Based on the summaries of the performance indicators in Table 4 and Table 5, the addition of synthetic training samples from the FS-GAN did not equate to using a real training dataset of equal size with respect to the performance of the proposed CNN. Furthermore, the inclusion of synthetic training samples in the training dataset decreased the model performance when compared to the peak performance established using a training dataset containing only real samples. Therefore, though the FS-GAN is capable of generating high-fidelity images from a small number of samples, the relative diversity of these generated samples is low, resulting in reduced generalization and performance when implemented to train the proposed CNN. This low diversity of synthetic samples is further evident when the density and radius of the training dataset clusters are analyzed. Figure 10 and Figure 11 depict the trend of the density and radius of the training dataset with increasing amounts of training samples for different real training data ratios. For real datasets, it can be observed that the density of the dataset is relatively low, suggesting a high distribution of the representation of the samples for all classes. In contrast, those training datasets with relatively high percentages of training samples that are synthetic have significantly higher densities, particularly those with 90% and 75% synthetic training samples (Scenarios 2–3). Though Scenarios 4–5 have relatively similar densities when compared to Scenario 1 which is trained using entirely real samples, there is a quantifiable difference in the space as demonstrated by Figure 11.

Though the densities are similar for Scenarios 2–4 when compared to Scenario 1, there is a prevalent difference in the radius of the clusters. From Figure 11, it can be observed that as the percentage of synthetic training samples is increased, the overall radius of the cluster decreases. Therefore, training datasets that contain a higher number of synthetic samples are characterized as having denser, less-volumetric clusters within the feature spaced when compared to clusters with higher levels of real samples present. Though the FS-GAN is capable of generating high-quality samples, from the analysis, the generator fails to diversify the samples resulting in a less generalized dataset. The result of training the proposed CNN using less diversified datasets is that the models will underperform and have an increased prevalence of overfitting when compared to training using entirely real samples. Moreover, this lack of generalization within the synthetic samples results in training clusters whose centroids are vastly different when compared to those clusters corresponding to the validation and testing datasets as summarized in Table 7. Comparing the centroid of the training clusters to the centroids of the validation and testing clusters, it can be observed that the distance between clusters is correlated to the ratio of real data used in the training datasets.

The overall trend when comparing the distance between training and validation clusters ($d_{TV}$) and training and testing clusters ($d_{TT}$) is that as the amount of synthetic samples increases, the distance between clusters increases. As the distance between the clusters increases, the likelihood that samples from the respective clusters will occupy the same space decreases, resulting in more distinct, less interactive clusters. For training datasets that contain a large amount of synthetic data (i.e., Scenario 1), it is observed that the addition of new training samples in the training dataset did not increase the validation and testing accuracy and overfitting remained prevalent. This observation can be explained by the distance of the training, validation, and testing clusters; since the distance between them is large, the samples do not occupy similar feature space and therefore the proposed CNN has difficulty classifying these images. Conversely, at higher ratios of real training data, the distance between the clusters is much lower which suggests that the clusters are more similar to each other and therefore occupy similar volumetric space within the provided feature space. As such, this is why the overfitting is reduced as more real samples are added to the training dataset and the overall performance indicators with respect to the validation and testing datasets improve.

## 6. Conclusions

In this paper, the feasibility of applying GANs to limited datasets within the SHM domain for multiclass damage detection of concrete structures is explored. Using images extracted from the SDNET 2018, an FS-GAN for each class of data is trained for the purposes of generating synthetic images to augment an existing real training database for CNN-based classification. An iterative process is applied by which the training dataset is progressively increased in size with both real and synthetic images and the proposed CNN is trained to classify between construction joints, cracks, pitting, and undamaged concrete surfaces. The accuracy, recall, precision, and F1 score for each iteration belonging to each training database configuration are calculated to determine the correlation between training dataset composition and classification performance. Centroidal-based clustering analysis is conducted for each iteration of the training dataset and the density and radius of the cluster as well as the distance between training and validation, and training and testing clusters. Though the FS-GAN is capable of generating high-quality images, it is concluded that the addition of synthetic images to an existing real training dataset did not significantly improve the performance of the proposed CNN network. Based on the analysis conducted, the following trends can be observed with respect to data augmentation of limited datasets using GANs for multiclass damage detection of concrete structures:

1. Training datasets comprised of both real samples and synthetic samples generated by the FS-GAN resulted in reduced performance of the proposed CNN when implemented to train the network. It is observed that as the ratio of the amount of synthetic data within the training dataset increased, the overall performance of the network decreased based on the chosen performance indicators. Comparing the real dataset (Scenario 1) to those incorporating synthetic images (Scenarios 2–5), it is determined that the performance of the trained CNN on the validation and testing dataset decreased on average by 10.6% and 7.4%.
2. For those scenarios that used synthetic training samples (Scenarios 2–5), the performance of the network decreased once synthetic samples are added to the training dataset. It is observed that the overall trend showed that for increasing ratios of synthetic samples within the dataset, the performance decrease of the proposed CNN dataset on only the real portion of the training data increased. On average, the performance of the proposed CNN decreased by −1.6% after the introduction of synthetic samples.
3. Those training datasets that contained synthetic samples has higher cluster densities and small cluster radius when compared to those that are comprised only of real samples. As the ratio of synthetic samples increased, the radius and density of the cluster decreased which infers the diversity of the dataset is becoming more homogeneous. Furthermore, the distances between the clusters belonging to the training, validation, and testing dataset increase with increasing synthetic samples which contributes to the inability of the proposed CNN to properly classify the validation and testing datasets as they do not occupy similar feature space.

Though the results summarized in this study demonstrate the potential ineffectiveness of GANs for limited-data domains, several factors are not explored in detail in this study. This study only analyzed one GAN and classifier model; therefore, in order to generalize the conclusions drawn from this study, additional GAN architectures and classifier models should be explored. Moreover, this study did not quantify the effect of the number and complexity of classes within the dataset on the performance of GANs for data augmentation. Lastly, this study did not address the class imbalance problem, which is a prevalent issue within the SHM community. Though prior research with GANs has studied the applicability of generative models for data augmentation of imbalanced datasets, there are no studies that investigate the use of GANs for limited unbalanced datasets. Future work should explore solving these existing challenges in addition to the development of GAN architecture which is capable of generating images with increased diversity and the application of Few-Shot Learning models for SHM problems.

## Figures and Tables

**Table 1:** Composition of dataset extracted from SDNET 2018 for GAN-based data augmentation.

Class	Construction Joint	Crack	Pitting	Undamaged
Training database	550	550	550	550
Validation database	40	40	40	40
Testing database	40	40	40	40

**Table 2:** Composition of training, validation and testing dataset for each training scenario.

Scenario #	Training—Real	Training—Synthetic	Validation	Testing
1	2200	0	160	160
2	1660	540	160	160
3	1100	1100	160	160
4	540	1660	160	160
5	200	2000	160	160

**Table 3:** Composition of training dataset for each iteration given a particular training scenario.

Iteration #	Scenario #									
	1		2		3		4		5	
	Real	Gen.	Real	Gen.	Real	Gen.	Real	Gen.	Real	Gen.
1	100	0	100	0	100	0	100	0	100	0
2	200	0	200	0	200	0	200	0	200	0
3	300	0	200	100	300	0	300	0	300	0
4	400	0	200	200	400	0	400	0	400	0
5	500	0	200	300	500	0	500	0	500	0
6	600	0	200	400	540	60	600	0	600	0
7	700	0	200	500	540	160	700	0	700	0
8	800	0	200	600	540	260	800	0	800	0
9	900	0	200	700	540	360	900	0	900	0
10	1000	0	200	800	540	460	1000	0	1000	0
11	1100	0	200	900	540	560	1100	0	1100	0
12	1200	0	200	1000	540	660	1100	100	1200	0
13	1300	0	200	1100	540	760	1100	200	1300	0
14	1400	0	200	1200	540	860	1100	300	1400	0
15	1500	0	200	1300	540	960	1100	400	1500	0
16	1600	0	200	1400	540	1060	1100	500	1600	0
17	1700	0	200	1500	540	1160	1100	600	1660	40
18	1800	0	200	1600	540	1260	1100	700	1660	140
19	1900	0	200	1700	540	1360	1100	800	1660	240
20	2000	0	200	1800	540	1460	1100	900	1660	340
21	2100	0	200	1900	540	1560	1100	1000	1660	440
22	2200	0	200	2000	540	1660	1100	1100	1660	540

**Table 4:** Comparison of performance indicators for training, validation, and testing datasets for different training dataset sizes.

Scenario	Dataset	Training Dataset Size							
		*n* = 100				*n* = 2200			
		mACC	mP	mR	mF1	mACC	mP	mR	mF1
1	Training	99.00	0.98	0.98	0.98	98.14	0.96	0.96	0.96
	Validation	74.38	0.48	0.49	0.48	89.38	0.79	0.79	0.79
	Testing	77.19	0.52	0.54	0.53	90.63	0.82	0.82	0.82
2	Training	99.00	0.98	0.98	0.98	99.93	1.00	1.00	1.00
	Validation	74.38	0.48	0.49	0.48	70.94	0.43	0.42	0.42
	Testing	77.19	0.52	0.54	0.53	75.63	0.51	0.51	0.51
3	Training	99.00	0.98	0.98	0.98	99.75	1.00	1.00	1.00
	Validation	74.38	0.48	0.49	0.48	80.31	0.63	0.63	0.63
	Testing	77.19	0.52	0.54	0.53	81.25	0.63	0.61	0.62
4	Training	99.00	0.98	0.98	0.98	99.23	0.98	0.99	0.98
	Validation	74.38	0.48	0.49	0.48	81.88	0.65	0.64	0.64
	Testing	77.19	0.52	0.54	0.53	88.13	0.77	0.77	0.77
5	Training	99.00	0.98	0.98	0.98	99.25	0.99	0.99	0.99
	Validation	74.38	0.48	0.49	0.48	81.88	0.66	0.64	0.65
	Testing	77.19	0.52	0.54	0.53	87.87	0.76	0.76	0.76

**Table 5:** Comparison of maximum mACC using real training data and average mACC using synthetic and real training data for validation and testing datasets.

Scenario	Dataset	Maximum mACC (%) (Real Data Only)	Average mACC (%) (Real and Synthetic Data)	Δ (%)
**2**	Validation	74.38	73.17	−1.21
	Testing	77.19	74.95	−2.24
**3**	Validation	79.69	78.86	−0.83
	Testing	83.91	80.69	−3.22
**4**	Validation	85.63	82.98	−2.65
	Testing	88.75	86.32	−2.43
**5**	Validation	86.25	84.92	−1.33
	Testing	88.59	89.48	+0.89

**Table 6:** Comparison of classification accuracy of different size testing datasets for Scenario 5.

Training Dataset Size	Accuracy (%)						Average (%)	SD (%)	Min (%)	Max (%)	Range (%)
	Testing Dataset Size										
	160	560	960	1360	1760	2160					
100	74.38	71.88	72.50	73.97	72.09	71.46	72.71	1.09	71.46	74.38	2.92
200	74.69	75.09	74.32	76.88	75.68	73.84	75.08	0.99	73.84	76.88	3.04
300	76.25	73.66	73.85	75.30	74.43	72.99	74.41	1.09	72.99	76.25	3.27
400	76.41	73.57	73.54	75.77	75.63	74.54	74.91	1.10	73.54	76.41	2.86
500	76.56	73.57	74.01	75.00	75.43	74.59	74.86	0.97	73.57	76.56	2.99
600	77.81	71.43	71.51	72.46	72.67	71.32	72.87	2.27	71.32	77.81	6.49
700	78.75	71.70	71.35	73.16	72.16	72.64	73.17	2.58	71.35	78.75	7.40
800	74.69	72.80	72.81	74.37	74.29	72.85	73.63	0.82	72.80	74.69	1.89
900	69.69	70.45	70.83	71.80	71.70	70.16	70.77	0.77	69.69	71.80	2.11
1000	78.13	74.11	74.74	76.66	76.37	76.07	76.01	1.31	74.11	78.13	4.02
1100	77.19	75.44	75.42	76.95	76.89	75.89	76.29	0.74	75.42	77.19	1.77
1200	76.88	72.95	73.44	75.41	75.34	74.21	74.70	1.33	72.95	76.88	3.93
1300	71.25	69.91	70.26	74.30	72.45	70.82	71.50	1.49	69.91	74.30	4.39
1400	75.94	69.29	69.70	70.37	70.54	69.65	70.91	2.29	69.29	75.94	6.65
1500	70.31	70.36	70.83	71.98	71.90	70.39	70.96	0.71	70.31	71.98	1.67
1600	74.38	72.14	73.12	72.65	68.86	72.53	72.28	1.69	68.86	74.38	5.52
1700	73.75	71.25	72.08	74.05	74.29	72.97	73.06	1.09	71.25	74.29	3.04
1800	72.19	69.82	71.77	73.13	72.98	71.67	71.93	1.09	69.82	73.13	3.30
1900	72.81	73.48	73.23	73.13	73.24	72.02	72.98	0.48	72.02	73.48	1.47
2000	73.75	71.43	73.49	73.49	72.62	71.76	72.76	0.90	71.43	73.75	2.32
2100	76.88	74.47	73.44	73.79	73.15	73.38	74.18	1.27	73.15	76.88	3.72
2200	75.63	72.14	72.97	73.46	73.58	72.69	73.41	1.10	72.14	75.63	3.48

**Table 7:** Distance between training and validation clusters and training and testing clusters for varying ratios of synthetic training data.

Scenario	$d_{\mathrm{TV}}\left(\mathrm{px}\right)$	$d_{\mathrm{TT}}\left(\mathrm{px}\right)$
Scenario 1	1.54	0.62
Scenario 2	5.08	4.21
Scenario 3	2.74	1.95
Scenario 4	1.39	2.04
Scenario 5	1.18	1.67

## Data Availability

Not applicable.

## Abbreviations

The following abbreviations are used in this manuscript:

AI	Artificial Intelligence
CNN	Convolutional Neural Network
DL	Deep Learning
GAN	Generative Adversarial Network
FS-GAN	Few Shot Generative Adversarial Network
FSL	Few-Shot Learning
mACC	Mean Accuracy
mF1	Mean F1 Score
ML	Machine Learning
mP	Mean Precision
mR	Mean Recall
SD	Standard Deviation
SHM	Structural Health Monitoring
